# Effects of Boric Acid and Storage Temperature on the Analysis of Microalbumin Using Aptasensor-Based Fluorescent Detection

**DOI:** 10.3390/bios12110915

**Published:** 2022-10-24

**Authors:** Chalermwoot Sompark, Wireeya Chawjiraphan, Manatsaphon Sukmak, Ubon Cha’on, Sirirat Anutrakulchai, Prapasiri Pongprayoon, Thitirat Putnin, Dechnarong Pimalai, Visarute Pinrod, Deanpen Japrung

**Affiliations:** 1 National Nanotechnology Center (NANOTEC), National Science and Technology Development Agency (NSTDA), Thailand Science Park, Pathumthani 12120, Thailand; 2 Postharvest and Processing Research and Development Division, Department of Agriculture, Bangkok 10900, Thailand; 3 Department of Biochemistry, Faculty of Medicine, Khon Kaen University, Khon Kaen 40002, Thailand; 4 Chronic Kidney Disease Prevention in the Northeast of Thailand (CKDNET), Khon Kaen University, Khon Kaen 40002, Thailand; 5 Department of Internal Medicine, Faculty of Medicine, Khon Kaen University, Khon Kaen 40002, Thailand; 6 Faculty of Science, Department of Chemistry, Kasetsart University, Chatuchak, Bangkok 10900, Thailand; 7 Center for Advance Studies in Nanotechnology for Chemical, Food and Agricultural Industries, KU Institute for Advance Studies, Kasetsart University, Bangkok 10900, Thailand

**Keywords:** boric acid, storage temperature, microalbumin, graphene oxide-based fluorescent detection

## Abstract

The instability of human serum albumin (HSA) in urine samples makes fresh urine a requirement for microalbumin analyses using immunoturbidimetry. Here, we determined the ability of an aptasensor-based fluorescent platform to detect microalbumin in old, boric acid-preserved urine samples. Our results show that the cleavage site of protease enzymes on urine albumin protein differed from the binding position of the aptamer on HSA protein, suggesting the aptasensor may be effective for albumin detection in non-fresh urine. Furthermore, the addition of boric acid in urine samples over a short term (at ambient temperature (T_a_) and 4 °C), long term (−20 and −80 °C), and following freeze–thawing (1–3 cycles) did not significantly affect albumin stability, as analyzed using the aptasensor. Therefore, boric acid stabilized has in urine stored over a short- and long-term. Thus, the aptasensor developed by us is applicable for HSA detection in boric acid-preserved urine that has been stored for 7-d at T_a_ and 4 °C, and in the long-term at −80 °C.

## 1. Introduction

Albumin is usually found in blood plasma and helps maintain the osmotic pressure between blood vessels and tissue [1]. It can also act as a carrier of materials necessary for controlling blood clotting [2]. Albuminuria is a pathological condition in which there is an abnormally high concentration of albumin in urine [3,4], which is a sign of liver cirrhosis in patients with chronic hepatitis and chronic kidney disease (CKD) [5,6]. The albumin concentration in the urine of healthy adults is <30 μg/mL, whereas in microalbuminuria, it ranges from 30 to 300 μg/mL. Albumin concentration is therefore one of the biomarkers for abnormal kidney function [7,8].

The standard tests for urine albumin detection in hospitals are immunological-based assays, such as immunonephelometry, radioimmunoassay, and immunoturbimetry assays [9]. These assays require anti-human albumin antibody to form an albumin–antibody binding complex, creating a turbidity signal; this signal is used to determine the concentration of urine albumin. Although immunological-based assays are highly sensitive, they require expensive and complex instruments [10], which are only installed in federal government hospitals and fully serviced private medical laboratories. In Thailand, when the screening and follow-up of diabetes mellitus and CKD patients in community hospitals and medical clinics are conducted, urine samples are immediately transported to hospitals/laboratories for albuminuria measurement. However, sample degradation occurs over the time it takes to transport the urine samples from the collection sites to hospitals, which may be located at long distances; this affects the results. Therefore, numerous studies have investigated urine preservation methods to prolong urinary albumin stability, such as the addition of protease inhibitors [11,12]. However, most of the well-known protease inhibitors (pepstatin and leupeptin) are peptides or proteins, which are affected on low urinary protein concentration analysis. Adding some acids to adjust urine pH and inhibit bacterial growth were also reported. Although, most of the acids, including hydrochloric, acetic acid and boric acid can denature urinary albumin, adding boric acid at certain concentrations (1.8–2.0%) can preserve the content of albumin, sugar, and blood cells. Therefore, boric acid has been used as a preservative agent to stabilize urinary albumin at 4 and 18 °C over 7 d and at −40 and −80 °C over 6 months [13,14]. However, the addition of preservatives increases costs and may interfere with other molecules of interest in the urine samples.

**Figure 1:**
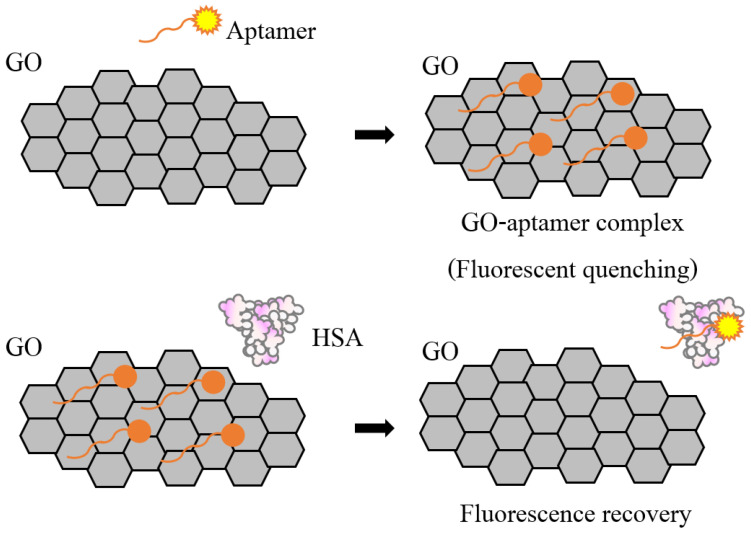
Schematic showing aptasensor technology for albumin detection used in this study.

A novel, sensitive detection method for urinary albumin was developed using graphene oxide-based fluorescent detection [15,16]. This platform uses an aptamer, a short single DNA with <100 nucleotides specifically bound to HSA, and the quenching property of graphene oxide to detect albumin in human serum and urine samples, as shown in Figure 1.

The simulation results indicated that the aptamer binding sites on the HSA protein were in subdomain IIIB [17]. Molecular dynamic simulation also revealed that the polar and charged amino acid residues play an important role in maintaining the affinity binding of the aptamer to lysine (K), arginine (R), histidine (H), glutamic (E), and aspartic (D) amino acid residues [17]. Therefore, the aim of this study was firstly to explore the efficiency of graphene oxide-based fluorescent detection for detecting urinary albumin that has been stabilized in boric acid-preserved urine under different storage conditions and temperatures, and secondly, to compare the efficiency of the developed method with the standard methods of detection used in hospitals.

## 2. Materials and Methods

### 2.1. Materials and Reagents

Graphene oxide (GO) was synthesized by a modified Hummers method as described in Jumpathong et al. (2016) [18]; a GO solution with a concentration of 5 mg/L was prepared by dissolving the GO powder in sterile, single distilled water. The characterization of GO was shown in the supporting information (Figures S1 and S2). The aptamer used here was an 87-nucleotide ssDNA (5’-Cy5-ATA CCA GCT TAT TCA ATT CCC CCG GCT TTG GTT TAG AGG TAG TTG CTC ATT ACT TGT ACG CTC CGG ATG AGA TAG TAA GTG CAA TCT-3’) [16] synthesized by Integrated DNA Technologies, Singapore. The urinary albumin analysis conditions were optimized according to a previous study [15,16]. Human serum albumin (HSA) was purchased from Sigma-Aldrich, St. Louis, MO, USA (CAS-No:70024-90-7). Boric acid powder (H_3_BO_3_) was purchased from Merck KGaA, Darmstadt, Germany (CAS-No:10043-35-3). Phosphate buffer saline (PBS) was obtained from Apsalagen, Bangkok, Thailand. All other reagents were analytical grade and used without further purification. Water was filtered through a Barnstead™ LabTower™ EDI Water Purification System (Thermo Scientific, Waltham, MA, USA) before use in all experiments.

### 2.2. Urine Sample Collection and Processing

**Figure 2:**
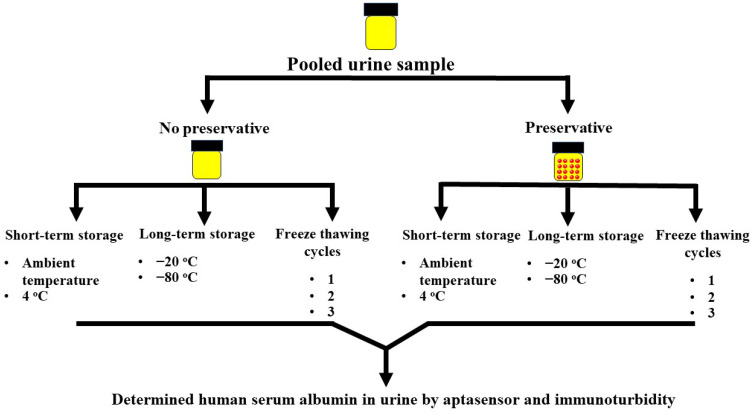
Schematic diagram showing the study design.

All urine samples were collected and studied under the ethical approval number, HE601035, from the office of the Khon Kaen University Ethics Committee in Human Research (Institutional Review Board number IRB00001189), Khon Kaen University, Thailand. Fourteen random spot urine samples were collected in urine collection cups. Then, 5 mL of urine sample was pipetted from each cup and pooled in sterile 50 mL Corning ™ polypropylene tubes. The pooled urine sample was divided into two tubes, and boric acid (2% *w*/*v*) was added to one tube. The aliquots were further subdivided to study the effects of temperature and storage conditions (over a short term at 4 °C and ambient temperature (T_a_, 25 °C)), long term at −20 and −80 °C, and following 1–3 freeze–thaw cycles (Figure 2). HSA concentrations in all samples were analyzed using graphene oxide-based fluorescent detection (Excitation at 630 nm and emission at 670 nm) and immunoturbidimetry.

### 2.3. In Silico Protease Digestion Study of Subdomain IIIB of the HSA Protein

**Figure 3:**
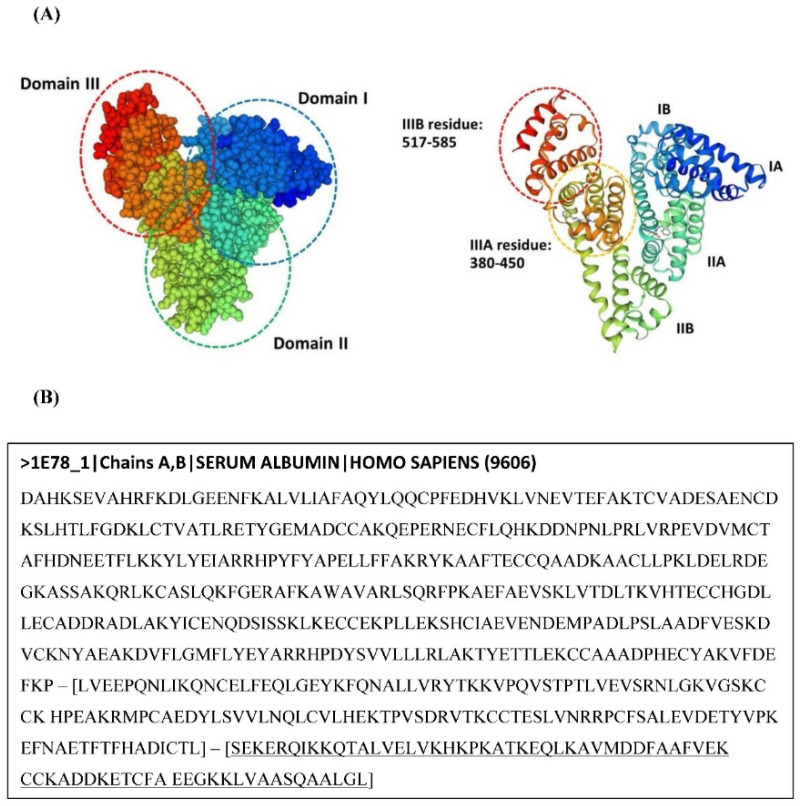
HSA structure (**A**) and HSA amino acid sequence with subdomain IIIB showing under the underlined sequence (**B**).

The results from our previous computer simulation study [17] indicated that the binding site of the aptamer on HSA protein was at subdomain IIIB. Thus, in this study, we used the PeptideCutter program (http://www.expasy.ch/tools/peptidecutter accessed on 14 September 2020) to predict the hydrolysis site of the HSA by protease enzymes, particularly peptidyl-Asp metalloendopeptidase (EC Number 3.4.24.33), which has been reported as a potential HSA hydrolysis enzyme in urine samples [11]. Full-length HSA amino acid sequences were downloaded from the Protein Data Bank (PDB ID: 1E78) (Figure 3).

### 2.4. Optimization of Aptamer Concentration

In sterile 500 μL Eppendorfs, 15 μL of 5 mg/L GO solution was mixed with 15 μL of H8 aptamer at various concentrations between 0.02 and 5 μM. The mixed solution was incubated at T_a_ in the dark for 5 min to allow the formation of the aptamer–GO complex and to quench the fluorescent signal. Subsequently, 20 μL of 30 μg/mL HSA was added to the solution, which was then incubated at T_a_ in the dark for 30 min. Subsequently, the solution volume was adjusted to 200 μL by the addition of PBS in the control group and 2% (*w*/*v*) boric–PBS buffer in the experimental group. The fluorescent signal from each was then measured using a Quantus^TM^ Fluorometer (Promega Corporation, Madison, WI, USA). The optimal aptamer concentration was taken as the concentration that completely quenched the fluorescence intensity.

### 2.5. Performance of HSA Analysis Using GO-Based Fluorescent Assay

The binding affinity of GO-based fluorescent detection was studied in PBS and 2% (*w*/*v*) boric-PBS following the modified protocol from our previous study [15,16], as described in the previous section. Then, the percentage fluorescence response (%F) was calculated using Equation (1). The %F values were plotted against the HSA concentrations (μg/mL), and linear regression was applied in PBS and boric-PBS buffer.

%F = [(F_ob_ − F_min_) × 100]/[(F_ap_ − F_min_)] (1)

where F_ob_ is the fluorescence intensity at various HSA concentrations (20–3000 μg/mL), F_min_ is the fluorescence intensity of the aptamer-bound GO, which is the negative control in this study, and F_ap_ is the maximum fluorescence intensity of 1 μM aptamer.

### 2.6. Determination of HSA in Pooled Urine Samples Using the Standard Method

The concentration of HSA in pooled urine samples was determined by an immunoturbidity assay using a Cobas 8000 c602 analyzer (Roche Diagnostics GmbH, Mannheim, Germany), which is the standard method used at the Srinagarind Hospital, Khon Kaen University. The same samples were also aliquoted to determine HSA concentration using a GO-based fluorescent assay as described in the previous section, with minor modifications. Here, instead of the stock solutions, 100 µL of pooled urine samples was used. Then, the mean percent change of HSA by immunoturbidity assay and the GO-based fluorescent assay were calculated using Equation (2).

Mean percent change of HSA = [(HSA_0_ − HSA_t_) × 100/HSA_0_] (2)

where HSA_0_ is the HSA concentration (μg/mL) from the pooled fresh urine sample and HSA_t_ is the HSA concentration from the pooled urine sample stored at different temperatures and freeze–thawing cycles.

### 2.7. Data Analysis

Pearson’s correlation coefficient (r) and *p*-value were calculated using SPSS for Windows (version 16.0). Tukey’s honest significant difference test was used to derive statistical differences between the control and testing conditions.

## 3. Results and Discussions

### 3.1. In Silico Protease Digestion of HSA Subdomain IIIB

In our previous study [17], the result showed that the binding site of the aptamer on HSA was the subdomain IIIB, which is the region starting from serine (S517) to lysine (L585). The lysine residues in this region were shown to play a role in the binding of the HSA protein and the aptamer, and the polar and charged amino acid residues helped to maintain the binding of HSA and the aptamer. The possible binding sites of HSA and aptamers were Lys (K), Arg (R), His (H), Glu (E), and Asp (D), as shown in Table 1 (underlined).

The results of the in silico protease digestion of subdomain IIIB using the PeptideCutter program are shown in Table 2; the Asp-N endopeptidase specifically cleaves bonds with the N-terminal side of Asp (D) at position P1’ [19]. The results from our in silico study showed that the selected protease enzyme digested the HSA subdomain IIIB at the amino acid positions 548, 549, 561, and 562, which are not aptamer-binding positions. It is possible that the subdomain IIIB of HSA was degraded into three fragments with weights ranging from 1331.61 to 3717.15 Da. Although the in vivo study of possible mechanisms by enzymatic degradation explained that the HSA possibly degraded from the acid endo protease within lysosomes at low pH (pH 4.0), the FcRn molecules, which are transcytosis receptors, could protect HSA from the acid endo protease degradation by binding with the HSA sequence. HSA is mainly making hydrophobic interactions with residues of FcRn at the amino acid sequence 422–551 (T422, V426, L460, L463, H464, T467, F507, F509, and F551). The domain III of HSA plays an essential role for binding, but domain I is also necessary for the complex formation and stabilization of the interaction [20].

Interestingly, the cleavage site of the Asp-N endopeptidase was not the same binding site as that of the HSA and the aptamer. This suggests that the aptamer could potentially bind to HSA proteins even in their degraded forms in urine.

### 3.2. Characteristics and Performance of the GO-Based Fluorescent Assay

**Figure 4:**
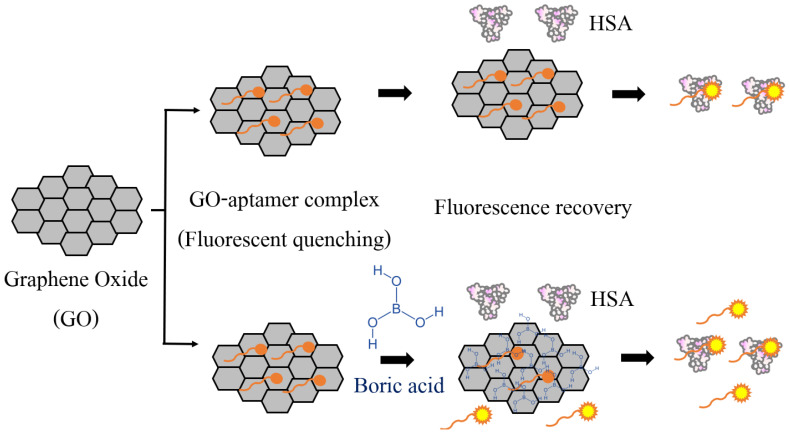
Schematic illustration of the graphene oxide-based fluorescent detection of human serum albumin (HSA) in boric acid-preserved urine samples.

A schematic illustration of the GO-based fluorescence detection of HSA in urine samples preserved in boric acid is shown in Figure 1 and Figure 4. In the absence of HSA, the aptamers are adsorbed onto the GO surface by π–π stacking, and the fluorescence of labeled dye is quenched via an energy transfer from the dyes to the GO. However, in the presence of HSA, the weak binding between the GO–aptamer complex allows the aptamer to fall off the GO surface and bind to the HSA, causing fluorescence recovery. It has been reported that there is a strong intermolecular hydrogen bonding between the hydroxyl groups in boric acid and the hydroxyl and carbonyl groups in GO [21]. Therefore, it is possible that boric acid competitively binds to GO once added to urine in the aptamer–GO complex, leading to a higher fluorescent recovery signal, compared with that in case of urine without the preservative. Our results supported this hypothesis, as the fluorescence intensity of the HSA analysis from PBS–boric buffer was higher than that from PBS without boric acid. The maximum fluorescent intensities were 8796 and 16,247 relative fluorescence units under PBS and PBS–boric buffer conditions, respectively.

**Figure 5:**
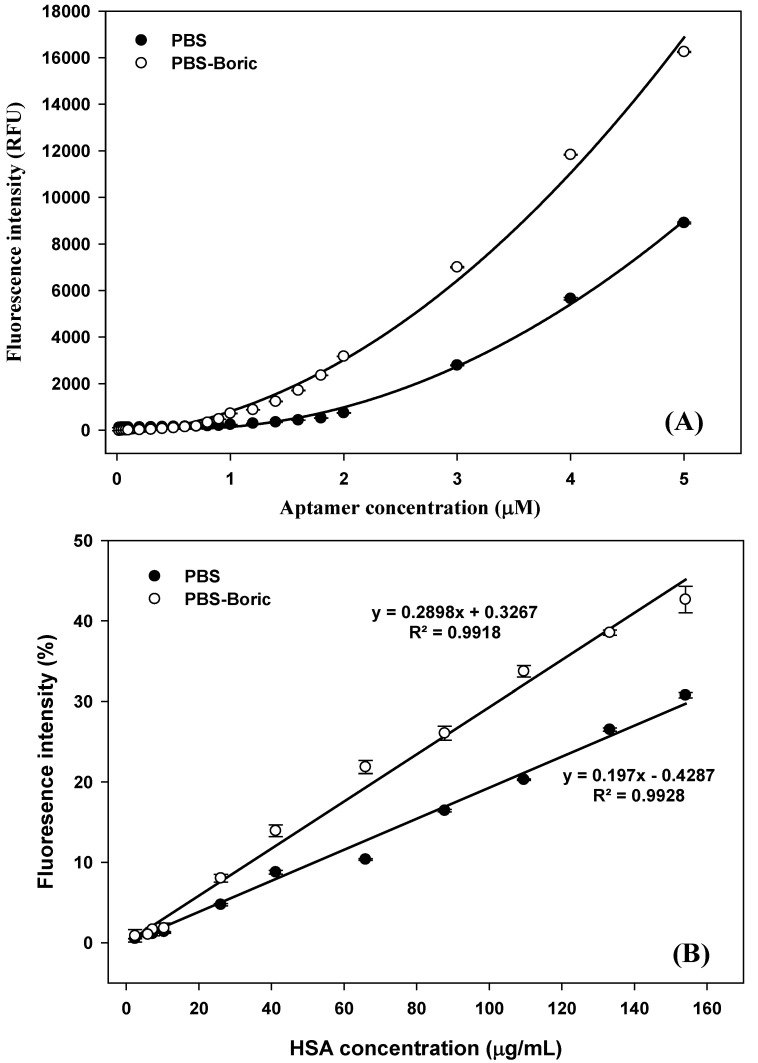
Fluorescence intensities of various concentrations of fluorescence-labeled H8 aptamer incubated with 5.0 mg/L GO in PBS buffer and PBS–boric buffer (**A**) and the calibration curve of fluorescence responses and HSA concentrations in PBS buffer and PBS–boric buffer determined using the developed aptasensor (**B**).

To determine the sensitivity and specificity of the developed aptasensor, the calibration curve of the fluorescence response was generated using the purified HSA in PBS buffer and PBS–boric buffer (Figure 5B), and the fluorescence images are shown in Figure S3. The fluorescence responses linearly correlated with HSA concentration between 2.42 and 154.05 μg/mL with a lower limit of detection (LOD) of 6.72 μg/mL (LOD = 3.3 × SD/slope, [15]) and a better response was observed in PBS–boric buffer than in PBS (R^2^ 0.9918 and 0.9928, respectively), as shown in Table S1. The sensitivity and specificity of the optimized aptasensor in this study were correlated with our previous studies [15,16]. In addition, it was lower cost compared with the existing methods used in the hospital and had a potential for point of care testing (POCT), as shown in the supporting information (Table S2).

### 3.3. Effects of Boric Acid Preservative, Storage Temperature, and Freeze–Thawing on Urine Microalbumin Stability

We evaluated the effects of the boric acid as a preservative, storage temperature, and freeze–thawing cycles on the urine microalbumin stability. Urine samples were collected from 14 randomly selected patients with early-stage CKD who presented with microalbuminuria (11.2–23.9 μg/mL) as per immunoturbidimetry assays. We also compared the mean percentage changes in HSA concentrations analyzed using our developed aptasensor and the immunoturbidimetry assay. The acceptable limits of variance for HSA analysis were set at ±10%, as reported by Giesen and Lieske (2016) [13].

#### 3.3.1. Effect of Short-Term Storage on Urine Microalbumin Stability

**Figure 6:**
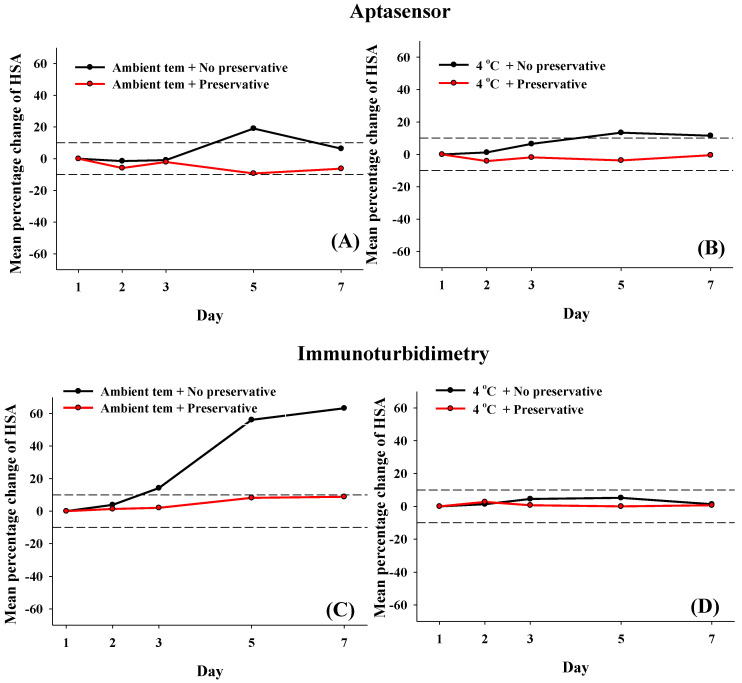
Mean percentage change in microalbumin analyzed by GO-based fluorescent detection (**A**,**B**) and immunoturbidimetry (**C**,**D**) with and without 2% (*w*/*v*) boric acid over a short term (1, 2, 3, 5 and 7 d). Dashed lines indicate ±10% change from the initial day 0 value.

The mean percentage change in HSA concentration was analyzed using our aptasensor and immunoturbidimetry, with and without 2% (*w*/*v*) boric acid, after short-term storage (1, 2, 3, 5, and 7 d) at T_a_ and 4 °C and compared (Figure 6). Under short-term storage (up to 7 d) at T_a_ and 4 °C, microalbumin remained stable, as determined by the aptasensor and immunoturbidimetry assays (Figure 6, red line). Smaller changes in microalbumin were observed at T_a_ when aptasensor assay was performed (over 3 d, −0.89% change) than with the immunoturbidimetry assay (over 2 d, 3.87% change), as shown in Figure 6A,C, black line. Based on the aptasensor readings, the mean percentage change in the microalbumin analyzed was −6.28% and −0.52% at T_a_ and 4 °C, respectively, and it remained stable for up to 7 d. In contrast, immunoturbidimetry assays showed mean percentage changes in microalbumin at 8.84% and −0.68% at T_a_ and 4 °C, respectively. Without preservative and at 4 °C, microalbumin changes were stable for only up to 3 days when analyzed by the aptasensor (6.45%) (Figure 6B, black line), but they were stable for up to 7 d when determined using the immunoturbidimetry assays (1.29%) (Figure 6D, black line).

#### 3.3.2. Effect of Long-Term Storage Conditions on the Stability of Microalbumin

**Figure 7:**
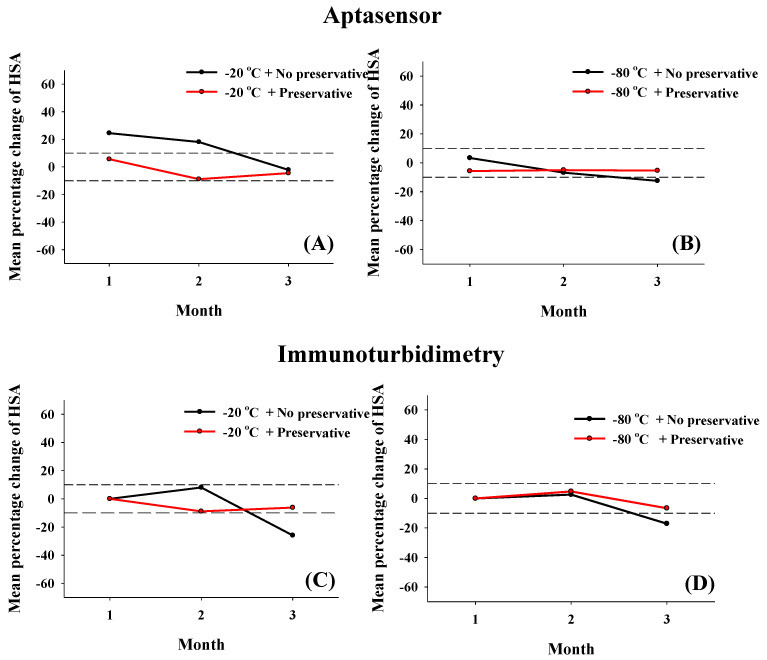
Mean percentage changes in microalbumin concentrations, as determined by GO-based fluorescent detection or by using the aptasensor (**A**,**B**) and by immunoturbidimetry (**C**,**D**) assays with and without 2% (*w*/*v*) boric acid following long-term storage (1, 2 and 3 months). Dashed lines indicate ±10% change from the initial day 0 value.

The mean percentage change in microalbumin concentrations, as analyzed using our aptasensor and immunoturbidimetry assay in the presence and absence of 2% (*w*/*v*) boric acid following long-term storage conditions (1, 2, and 3 months) at −20 and −80 °C, was determined (Figure 7). The addition of boric acid to urine samples stabilized the microalbumin content (analyzed using the aptasensor and by a standard method) for up to 3 months at −20 and −80 °C (Figure 7, red line). The mean percentage changes in microalbumin concentrations, as analyzed using the aptasensor, were −4.52% and −5.34% at −20 and −80 °C, respectively, over 3 months. Similarly, microalbumin concentrations, as analyzed by immunoturbidimetry, were determined to change by −6.28% and −6.64% at −20 and −80 °C, respectively. These values were >10% higher than the mean percentage microalbumin changes in urine samples that did not contain the preservative, stored at −80 °C for 3 months, based on both detection methods (Figure 7A,C, black line). However, at 2 months, the results obtained from both assays were similar (Figure 7B,D, black line).

#### 3.3.3. Effect of the Freeze–Thawing Cycle on the Stability of Urine Microalbumin

**Figure 8:**
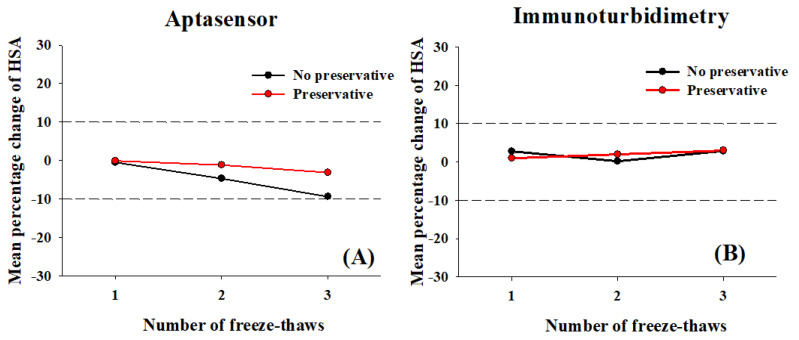
Mean percentage change in microalbumin concentrations, as determined by GO-based fluorescent detection or using the aptasensor (**A**) and by immunoturbidimetry (**B**) assays with and without 2% (*w*/*v*) boric acid following freeze–thaw cycles (1, 2, and 3 cycles). Dashed lines indicate ±10% change from the initial day 0 value.

Microalbumin was found to be stable in fresh urine samples with and without boric acid. Our study showed that the addition of boric acid to urine samples stabilized microalbumin during freeze–thaw cycles, with percentage changes of 2.86% and −3.09%, as determined using the aptasensor and standard assay, respectively (Figure 8, red line). In contrast, in non-preserved urine, the percentage change in microalbumin concentrations rapidly dropped (−9.36%) during three freeze–thaw cycles, as determined using the aptasensor; nevertheless, the microalbumin concentration remained at an acceptable value (<10%) (Figure 8A, black line).

## 4. Conclusions

This study demonstrates the effects of boric acid and storage temperature on the stability of urine microalbumin using a graphene oxide-based fluorescent detection method or an aptasensor and the standard immunoturbidimetry assay. We found that boric acid helped maintain the stability of HSA in urine following short- and long-term storage. Our results also suggested that the aptasensor developed in this study could effectively be used to detect HSA in urine samples preserved using boric acid and stored at T_a_ and 4 °C for up to 7 d, and at −80 °C for over 12 months. The findings of this study could be applied to albumin detection in urine samples collected from remote areas or from areas situated far away from well-equipped hospitals or medical technology laboratory services, thereby increasing the scope of diagnosis via HSA detection beyond the presently available methods.

## Figures and Tables

**Table 1:** The amino acid residues of the HSA subdomain IIIB (S517-L585), binding site of the aptamer on HSA, and the cleavage sites of the Asp-N endopeptidase (aspartyl protease).

HSA subdomain IIIB residue	SEKERQIKKQTALVELVKHKPKATKEQLKAVMDDFAAFVEKCCKADDKETCFAEEGKKLVAASQAALGL
Aptamer-binding position	SEKERQIKKQTALVELVKHKPKATKEQLKAVMDDFAAFVEKCCKADDKETCFAEEGKKLVAASQAALGL
Asp-N endopeptidase cleavage	SEKERQIKKQTALVELVKHKPKATKEQLKAVM***/***D***/***DFAAFVEKCCKA***/***D***/***DKETCFAEEGKKLVAASQAALGL

The underlined sequences are the binding sites of HSA and H8 aptamers. The symbols (***/***) represent the cleavage sites of Asp-N endopeptidase (aspartyl protease).

**Table 2:** The potential protease cleavage fragments of HSA subdomain IIIB.

Possible Protease	Cleavage Rules	No. of Cleavages	Position of Cleavage Site	Peptide Mass (Dalton)	Peptide Fragment
Asp-N endopeptidase	N-terminal side of D	4	548, 549, 561, 562	3717.15,1331.61, 2379.22	(1) SEKERQIKKQTALVELVKHK PKATKEQLKAVM (2) DFAAFVEKCCKA (3) DKETCFAEEGKKLVAASQAA LGL

## Data Availability

Not applicable.

## Supplementary Materials

The following supporting information can be downloaded at: https://www.mdpi.com/article/10.3390/bios12110915/s1, Figure S1: Transmission electron microscopy (JEM-2100Plus, JEOL, USA) image of synthesized GO used in this study; Figure S2: UV spectra of graphene oxide was recorded in the wavelenge of 200–500 nm using PowerWave XS2. The spectrum of graphene oxide has an absorption peak at 230 nm which is attributed to p-p* transition of remaining sp2 C=C bonds; Figure S3: Florescence images of the vial containing aptasensor solution with HSA concentration of 0 μg/mL (tube 1), 2 μg/mL (tube 2), 80 μg/mL (tube 3), 150 μg/mL (tube 4) and aptamer labeled with Cy5 fluorescence dye (positive control, tube 5) in PBS with boric acid (A) and without boric acid (B); Table S1: LOD and LOQ calculation; Table S2: Performance of existing methods use in hospital and the developed aptasensor (this study).
