# Supplementary material for: Effects of Boric Acid and Storage Temperature on the Analysis of Microalbumin Using Aptasensor-Based Fluorescent Detection
Source: Biosensors (Basel). 2022 Oct 24;12(11):915. doi: 10.3390/bios12110915 (PMC9687920; doi:10.3390/bios12110915)
Supplement: Supplementary file 1 [file biosensors-12-00915-s001.zip › biosensors-1945440-supplementary.pdf]

Supporting information

Effects of boric acid and storage temperature on the analysis of microalbumin using aptasensor based fluorescent detection

Chalermwoot Sompark[1,2], Wireeya Chawjiraphan[1], Manatsaphon Sukmak[3,5], Ubon Cha'on[3,5], Sirirat Anutrakulchai[4,5], Prapasiri Pongprayoon[6,7], Thitirat Putnin[1], Dechnarong Pimalai[1], Visarute Pinrod[1], Deanpen Japrung[1*]

[1] National Nanotechnology Center (NANOTEC), National Science and Technology Development Agency (NSTDA), Thailand Science Park, Pathumthani, Thailand
[2] Postharvest and Processing Research and Development Division, Department of Agriculture, Bangkok, Thailand
[3] Department of Biochemistry, Faculty of Medicine, Khon Kaen University, Khon Kaen, Thailand
[4] Department of Internal Medicine, Faculty of Medicine, Khon Kaen University, Khon Kaen, Thailand
[5] Chronic Kidney Disease Prevention in the Northeast of Thailand (CKDNET), Khon Kaen University, Khon Kaen, Thailand
[6] Faculty of Science, Department of Chemistry, Kasetsart University, Chatuchak, Bangkok, Thailand
[7] Center for Advance Studies in Nanotechnology for Chemical, Food and Agricultural Industries, KU Institute for Advance Studies, Kasetsart University, Bangkok, Thailand

* Correspondence: deanpen@nanotec.or.th; Tel.: +66 117 6665

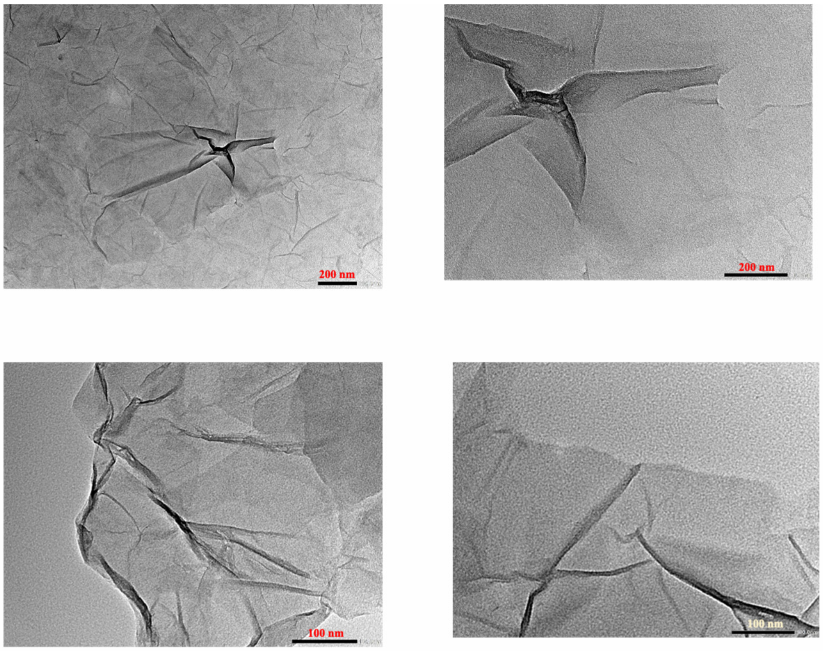

Figure S1. Transmission electron microscopy (JEM-2100Plus, JEOL, USA) image of synthesized GO used in this study.

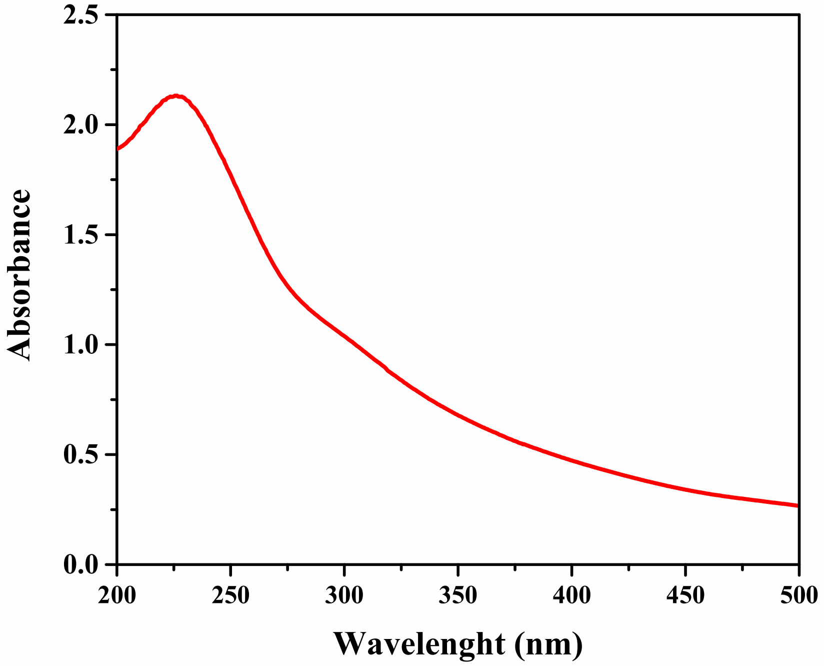

Figure S2. UV spectra of graphene oxide was recorded in the wavelenge of 200-500 nm using PowerWave XS2. The spectrum of graphene oxide has an absorption peak at 230 nm which is attributed to p-p* transition of remaining sp^2 C=C bonds.

(A). (B).

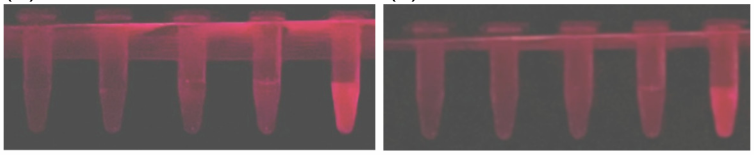

1. 2. 3. 4. 5. 1. 2. 3. 4. 5

Figure S3. Florescence images of the vial containing aptasensor solution with HSA concentration of 0 μg/mL (tube 1), 2 μg/mL (tube 2), 80 μg/mL (tube 3), 150 μg/mL (tube 4) and aptamer labeled with Cy5 fluorescence dye (positive control, tube 5) in PBS with boric acid (A) and without boric acid (B).

Table S1. LOD and LOQ calculation

Items	Condition with PBS	Condition with PBS and Boric
Equation	y = 0.197x - 0.4287, R^2=0.9928	y = 0.2898x + 0.3267, R^2=0.9918
Slope	0.197	0.2898
SE of intercept	0.441234	0.68914
LOD (μg/mL)	6.72	7.134
LOQ (μg/mL)	22.40	23.78

Table S2. Performance of existing methods use in hospital and the developed aptasensor (this study).

Comparing items	Immunoturbidimetry (Hospital use)	Lateral flow immunoassay (Hospital use)	Aptasensor (This study)
LOD or cut off	6 μg/mL (LOD)	30-50 μg/mL (cut off)	6.72 μg/mL
Assay type	Quantitative	Qualitative	Quantitative
Targeting ligand	Antibody	Antibody	Aptamer
Assay time (min)	5-10	5-10	30
POCT	No	Yes	Yes
Cost (USD)	2	5-10	0.3
